# Addressing a Flat‐Out Problem: Environmental DNA (eDNA) Exposes Silent Infestations of *Acropora* ‐Eating Flatworms ( *Prosthiostomum acroporae* ) in Coral Aquaculture

**DOI:** 10.1002/ece3.73386

**Published:** 2026-04-17

**Authors:** Clare M. Grimm, Jonathan Barton, David G. Bourne, Yui Sato, Jason Doyle

**Affiliations:** ^1^ Australian Institute of Marine Science Townsville Queensland Australia; ^2^ National Sea Simulator, AIMS Townsville Queensland Australia; ^3^ College of Science and Engineering James Cook University Douglas Queensland Australia; ^4^ AIMS@JCU, Division of Research and Innovation James Cook University Townsville Queensland Australia

**Keywords:** *Acropora*, *Acropora*‐eating flatworms, Australia, biomonitoring, coral, droplet digital PCR, environmental DNA, genetics

## Abstract

Captive culturing of coral is required to supply a burgeoning aquarium trade and potentially supply populations to replenish reefs that have been degraded globally due to anthropogenic impacts. High‐density coral propagation can be compromised by pests that reduce broodstock and offspring health. *Acropora*‐eating flatworms (*Prosthiostomum acroporae*) are parasitic polyclads that often evade visual detection and feed on tissues of branching coral species of the genus *Acropora*, which are key targets for coral aquaculture production. We developed a highly sensitive and specific droplet digital PCR (ddPCR) assay to detect environmental DNA (eDNA) of *P. acroporae* from water samples, with the limit of detection of 2.3 copies of the mitochondrial cytochrome c oxidase subunit I gene per ddPCR reaction. Validation was performed using water samples of aquaria holding *Acropora* corals suspected to be affected by *P. acroporae,* and the sequence identity of ddPCR amplicons confirmed by Sanger sequencing. *P. acroporae* eDNA was detected in some cases without visual confirmation of *P. acroporae*, indicating this assay can detect early infestations of *P. acroporae* that are not evident through visual monitoring. This study demonstrates that the ddPCR assay can be a valuable tool for monitoring pests in coral aquaculture systems and subclinical detection of parasitic infestations.

## Graphical abstract

Captive coral aquaculture is increasingly important for both the aquarium trade and reef restoration, but production can be undermined by hard‐to‐detect pests such as the *Acropora*‐eating flatworm *Prosthiostomum acroporae*. We developed and validated a highly sensitive ddPCR assay capable of detecting *P. acroporae* eDNA from aquarium water, even in cases where visual signs of infestation were absent. This tool enables early, subclinical detection of parasitic flatworms and provides a valuable genetic monitoring method for improving coral aquaculture health and biosecurity.

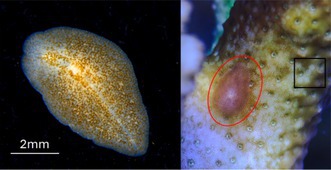

## 1 Introduction

**FIGURE 1:**
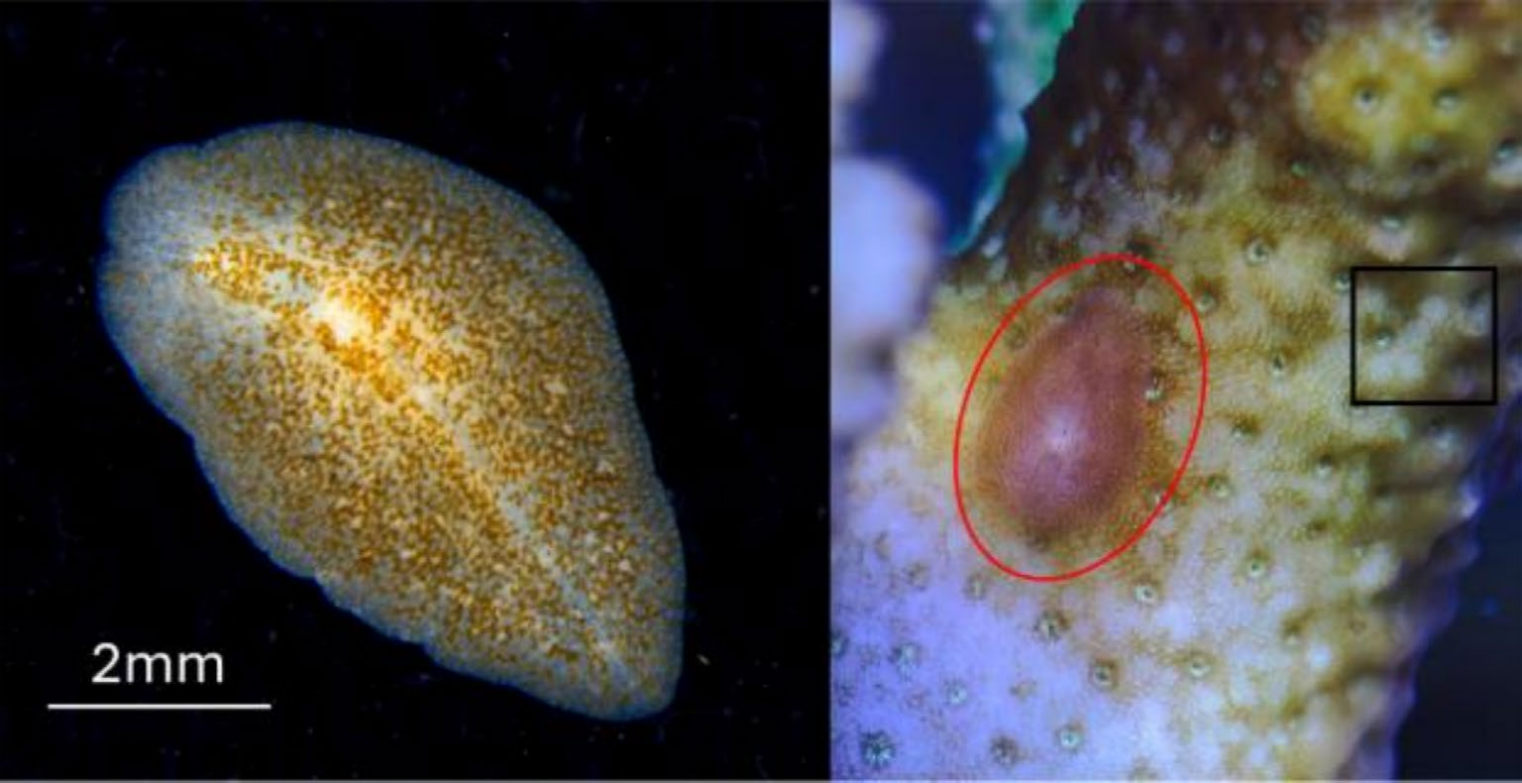
*Prosthiostomum acroporae*. Left panel is an isolated individual collected from an *Acropora* sp. host coral. Right panel demonstrates the cryptic camouflage of *P. acroporae* (inside red oval) whilst feeding on the *Acropora* sp. host coral. Example of feeding scar on *Acropora* sp. host by *P. acroporae* shown inside black box. Photo credit: Jonathan Barton.

Land‐based, or ex situ, coral nurseries and breeding practices (hereby defined as coral aquaculture) are growing to meet the demand for reef restoration initiatives to supply corals that can be out‐planted onto degraded reefs (Howlett et al. 2021; Ridlon et al. 2023) and to reduce the pressure on natural coral stocks from harvesting for the marine ornamental trade (Palmtag 2017). Coral aquaculture practices, however, face similar challenges to ones that plague other aquaculture ventures, including the proliferation of pests that can undermine coral production (MacAulay et al. 2022). For example, *Acropora*‐eating flatworms (AEFW), *Prosthiostomum acroporae*, are parasitic species of polyclad flatworms that feed on branching coral species of the genus *Acropora* and can persist as a prolific pest in coral aquaria (Figure 1; Litvaitis et al. 2019; Rawlinson et al. 2011). Infestations of these cryptic flatworms on captive *Acropora* were first observed by coral hobbyists (Barton et al. 2019; Nosratpour 2008), but have since been considered as a potentially serious problem to large‐scale coral aquaculture facilities as it undermines the health of captive corals and often evades visual detection and eradication (Barton, Bourne, et al. 2020; Barton, Humphrey, et al. 2020; Barton et al. 2019). *P. acroporae* have high fecundity, camouflaged nature, and complex life history (e.g., intracapsular larva). Tissue consumption by *P. acroporae* can lead to irreversible tissue damage of *Acropora* hosts and ultimately colony mortality if left unchecked (Barton et al. 2019; Hume et al. 2014; Rawlinson et al. 2011).

Coral pests, such as *P. acroporae*, are often microscopic, or otherwise difficult to detect. As such, clinical signs of infestations within aquaculture systems are often observed in late stages where delayed treatment options may become inadequate (Bass et al. 2015). Traditional aquaculture pest diagnostics, such as histopathology, culture work, genetic screening, and morphological identification, are often costly and time‐ and labour‐intensive, lack detection sensitivity unless a large number of samples are processed, or only detect the parasite when the culture has grown to an epizootic outbreak (Adrian‐Kalchhauser and Burkhardt‐Holm 2016; Eble et al. 2020; Gomes et al. 2017). A viable alternative to conventional pest monitoring can be provided by environmental DNA (eDNA): the sloughed or excreted genetic materials within environmental samples (i.e., water, sediment, soil), which can be detected without visual identification of the biological source (Goldberg et al. 2016; Thomsen and Willerslev 2015). This eDNA sampling approach has quickly evolved into a powerful diagnostic tool for detection of pests in a range of aquaculture species and systems. Applications include the detection of invasive Mediterranean fanworm ( *Sabella spallanzanii* ) in inshore mariculture mussel farms (Brand et al. 2022), the protozoan parasite (*Chilodonella hexasticha*) in inland barramundi ( *Lates calcarifer* ) farms (Gomes et al. 2017), and ectoparasitic monogenean flukes (*Dactylogyrus* spp.) in ornamental fish shipments (Trujillo‐González et al. 2019). However, eDNA‐based parasite detection has not been explored in coral aquaculture and developing biosecurity tools early in its development is advantageous for the growth of the industry.

Real‐time PCR (qPCR) has been widely used in aquaculture to detect a range of target species from environmental DNA (eDNA) samples (Gomes et al. 2017; Trujillo‐González et al. 2019). More recently, droplet digital PCR (ddPCR) has emerged as a powerful alternative, enabling absolute quantification of DNA without the need for standard curves (Hindson et al. 2011). This technology partitions a single PCR reaction into approximately 20,000 nano‐sized water–oil droplets, allowing individual amplification events to occur independently and separating positive and negative droplets by end‐point fluorescence reading (Hindson et al. 2011). Comparative studies have demonstrated several advantages of ddPCR over qPCR, including improved sensitivity and precision at low target concentrations, reduced susceptibility to user‐associated variability, and greater tolerance to PCR inhibitors (McNair et al. 2025; Nathan et al. 2014). Given the cryptic life history of *P. acroporae* and the presence of potential PCR inhibitors in coral aquaculture systems, such as coral tissue and mucus (Pratte and Kellogg 2021; Weber et al. 2017), ddPCR represents a promising platform for the development of a parasite‐specific eDNA assay. Accordingly, the objectives of this study were to (1) develop a species‐specific ddPCR assay for the *Acropora*‐eating flatworm, *P. acroporae*; (2) validate assay specificity and sensitivity through in silico and in vitro testing; and (3) evaluate its applicability within aquaculture settings using water samples collected from coral propagation systems.

## 2 Materials and Methods

### 2.1 Tissue Sample Collection, Primer Design, and In Silico Validation

*Acropora*‐eating flatworms identified as *Prosthiostomum acroporae*, were collected in 2021 from *Acropora millepora* coral hosts originating from Martin Reef and Falcon Reef within the Great Barrier Reef Marine Park (Table 1) (GBRMPA Permit G21/38062.1). Additional specimens of non‐targeted organisms, including commonly occurring aquarium species and platyhelminth flatworms were collected from the Australian Institute of Marine Science National Sea Simulator facility in August 2022 and Heron Island, Australia (23°26.5′ S, 151°54.4′ E) (Desbiens et al. 2023) in April 2023, respectively (Appendix S1). DNA from tissues of each specimen were extracted using a Qiagen Blood and Tissue kit, following the manufacturer's protocols with a minor modification—the specimen was added to Qiagen Buffer ATL (180 μL) and proteinase K (20 μL) and incubated overnight at 56°C with constant rotation to ensure thorough cell lysis.

**TABLE 1:** *Prosthiostomum acroporae* specimens for AEFW primer and probe design.

Specimen ID	GenBank accession	Description^a^	Species	Coral host	Origin
1	PV382155	Large individual	*Prosthiostomum acroporae*	*Acropora millepora*	Martin Reef 14°45.9′ S 145°21.3′ E
2	PV382156	Small individual			
3	PV382157	Egg cluster			
4	PV382158	Large individual	*Prosthiostomum acroporae*	*Acropora millepora*	Falcon Reef 18°46.5′ S 146°33.1′ E
5	PV382159	Small individual			
6	PV382160	Small individual			

^a^ Large individuals are approximately 4–6 mm, with small individuals 2–3 mm in length. Egg clusters contain approximately 30–40 eggs, with each egg approximately 0.4 mm diameter.

For designing ddPCR primers targeting the mitochondrial cytochrome oxidase subunit I (mtCOI) gene, template sequences of ~700 bp were generated from six *P. acroporae* specimens (Table 1) with the broad range mtCOI primers LCO1490 and HCO2198 (Folmer et al. 1994), following the PCR protocol specified in Folmer et al. (1994). PCR amplicons were purified and Sanger sequenced by Macrogen (South Korea). Forward and reverse sequences of the mtCOI gene from the specimens were trimmed to remove low‐quality and primer‐associated bases and then assembled to a sequence contig per sample. Contig sequences were aligned using the Muscle Alignment extension in Geneious Prime (v.2022.1.1 https://www.geneious.com/) to obtain a *P. acroporae* consensus sequence allowing base ambiguities. This consensus sequence was used to design forward and reverse ddPCR primers and a hydrolysis TaqMan probe with the following constraints: (1) melting temperature: 55°C–65°C with < 5°C difference between paired primers, (2) G/C content: 40%–80%, (3) primer and probe length: 15–30 bp, (4) amplicon size: 60–300 bp, and (5) non target sequences must have four or more mismatches to primer sequence including at least one mis‐matches within five base pairs of the 3′ end of the primer. Candidate primers and probes were assessed for possible secondary structure or duplex formation in silico using the IDT OligoAnalyzer Tool (https://sg.idtdna.com/calc/analyzer) and for potential binding against all non‐target metazoan species to avoid off‐target PCR amplification using PrimerBLAST (Ye et al. 2012) and NCBI nucleotide BLAST (blastn) tool (Altschul et al. 1997). The primer pair and probe that successfully met all the in silico criteria were synthesized with HPLC purification (Merck, Australia).

### 2.2 Droplet Digital PCR Assay Optimization

Digital droplet PCR (ddPCR) enables the accurate and sensitive distinction of directly amplified products through the nano‐partitioning of PCR reactions. Quantification occurs by distinguishing fluorescent signal intensities between template‐present (positive) and template‐absent (negative) droplets (Hindson et al. 2011). High signal separation between positive and negative droplets is therefore desirable for the clear definition of a threshold to identify positive detection. To optimize annealing/extension temperature and primer/probe concentrations for the ddPCR assay, a matrix of conditions was tested using signal‐to‐noise ratio and coefficient of variation (%CV) of the positive droplet fluorescence to optimize annealing/extension temperature and primer/probe concentrations for the ddPCR assay. Forward and reverse primers were tested at 400, 600, and 900 nM, and probes at 100 and 250 nM. All ddPCR was performed using the Bio‐Rad QX200 system, starting with an automated droplet generator using Droplet Generation Oil for Probes (Bio‐Rad). Plates containing ddPCR droplets were sealed with pierceable foil (Bio‐Rad) and run on a Bio‐Rad C1000 Thermal Cycler. The two‐step thermocycling protocol included a combined annealing/extension step, tested in duplicate from 57°C to 62°C in 1°C increments, followed by a denaturing step at 94°C for 40 cycles and finished with 10 min of enzyme deactivation at 98°C. Testing of different primer concentrations, probe concentrations and annealing temperatures was conducted on genomic DNA extract of *P. acroporae* (standardized to 15 pg/μL) and was run simultaneously in a 96‐well PCR plate to minimize potential run‐to‐run biases. Droplets were read on a QX200 droplet reader (Bio‐Rad), and signal profiles were analyzed using Bio‐Rad QuantaSoft Analysis Pro software version 1.0.596.05.25.

### 2.3 Sensitivity and Specificity of the AEFW ddPCR Assay

A 5‐fold dilution series of a *P. acroporae* genomic DNA (gDNA) extract was prepared producing template DNA concentrations ranging from 62 pg down to 0.004 pg per reaction. A total of eight replicates were conducted at each test dilution and negative controls were included. The limit of detection (LOD) was assessed as the lowest DNA input amount showing 100% positive amplification across all technical replicates (De Brauwer et al. 2023). To determine the limit of quantification (LOQ), the coefficient of variation (%CV) was calculated for each gDNA concentration, and the LOQ was defined as the lowest standard concentration with a %CV below 35% (Klymus et al. 2020). Quantitative linearity of the sensitivity data was evaluated by plotting the log10‐transfored copy concentration measurements against the log10‐transporded input DNA concentration and fitting a linear regression (Scriver et al. 2024; Zhao et al. 2016).

The specificity of the *P. Acroporae* ddPCR assay was tested using gDNA extracted from tissues of the non‐target species (Appendix S1), which include related polyclad flatworms and are commonly associated with aquarium invertebrates. The gDNA of non‐target species was quantified using a Qubit HS dsDNA kit (Thermo Fisher, Australia), and gDNA concentrations were adjusted for final concentrations of 0.1–0.5 ng/μL. Each specimen was tested in duplicate, with the ddPCR assay established above.

### 2.4 Applications of the eDNA‐ddPCR Assay to Coral Aquaculture

eDNA sampling was conducted in aquarium facilities within the National Sea Simulator (SeaSim) at the Australian Institute of Marine Science (AIMS), during the coral spawning event in November and December 2023, to test the assay performance in coral aquaculture settings. *Acropora millepora* colonies were collected in November from Falcon Island Reef (18°46′13.4″ S 146°31′56.7″ E). *Acropora spathulata* and *Acropora loripes* colonies were collected in December from Davies Reef (18°49′09.9″ S 147°38′57.9″ E). Colonies of all species were collected using a hammer and chisel on SCUBA (1–9 m depth; GBRMPA Permit G21/38062.1), fragmented to approximately 20 cm^2^, and transported to SeaSim, where they were held in outdoor aquaria. In November 2023, *A. millepora* colonies were housed in a tank connected to a partial recirculating aquaculture system. In December 2023, the *A. millepora* colonies were moved into a different tank within the same partial recirculating system, and *A. spathulata* colonies were placed in the tank where *A. millepora* colonies previously occupied. This partial recirculating system consisted of the two tanks holding the coral colonies and was connected by a shared sump with a 2 L/min top up of filtered seawater. *A. loripes* colonies were held in an isolated tank with 100% flow‐through filtered seawater at 200 L/h. All coral colonies were visually monitored for *P. acroporae* by aquarist husbandry technicians throughout the duration of their time in captivity, while control of *P. acroporae* on these colonies was not applied.

Tanks holding the coral colonies were sampled repeatedly, with the *A. millepora* tank sampled twice in November 2023 and twice in December 2023, and the *A. spathulata* and *A. loripes* tanks each sampled twice in December 2023 (total 8 sampling events; Table 4). At each sampling event, quadruplet replicate water samples (10 L each) for eDNA analysis were collected around coral colonies directly from tanks through an eDNA filter housing (Smith‐Root, USA) containing a 1.2 mm‐mesh mixed cellulose ester membrane of 47 mm in diameter (Rowe Scientific, Australia) using a portable eDNA sampling device (Grover‐Pro, Grover Scientific, Townsville Australia). Filter housings were placed on ice after sampling and within 2 h post sampling, the membrane filters were removed from the housings, folded carefully into eighths using bleach‐cleaned forceps and transferred into a 1.5 mL screw cap tube containing 540 mL of Qiagen Buffer ATL as a preservative. DNA was extracted from the filters using a Qiagen Dneasy Blood and Tissue kit on a Qiacube automated extraction instrument (Qiagen) with slight modifications from the manufacturer's protocol. Briefly, 60 μL of proteinase K was added to each screw‐cap tube, vortexed, and incubated overnight at 56°C with constant rotation. The full 600 μL volume was transferred to a 2 mL microtube, followed by 600 μL of Qiagen Buffer AL and a 30‐min incubation at 56°C with agitation. Next, 600 μL of 100% ethanol was added and mixed, then loaded onto a Qiagen spin column in three 600 μL steps. DNA was eluted in 50 μL of 10× diluted TE buffer. Negative controls were included at all levels of sample handling: “no‐template controls” (NTC), DNA‐extraction controls (blank filters extracted alongside samples) and “no‐coral controls” (filtered seawater) sampled in aquaculture facilities.

eDNA samples and controls were analyzed using the optimized ddPCR assay. To determine positive amplification, initial thresholds were set between the segregated groups of signal intensities derived from positive droplets and negative droplets, guided by the signal distribution of *P. acroporae* gDNA positive controls. After setting the initial thresholds across samples, the ddPCR profiles of each well was visually inspected and thresholds set midway between the positive control and the NTC to control false positives from weak signals and avoid false negatives from partial amplification. Samples were interpreted using a binary detection definition. Sampled tanks were considered positive when mean target concentrations were above the LOD or not statistically different from it. Tanks were considered negative when mean concentrations were below the LOD and statistically different from it. Statistical comparisons were performed using a two‐tailed *t*‐test comparing mean sample concentrations to the LOD, with significance assessed at a *p*‐value of 0.05. To confirm *P. acroporae* presence, positive eDNA samples were sequenced via Sanger sequencing (Macrogen Inc., South Korea). Sequences from eDNA positive samples were compared to gDNA sequences of *P. acroporae* (Table 1; Appendix S1) and *Prosthiostomum* spp. Sequences available from NCBI using Geneious Prime using MAFFT alignment v.7.490 (Katoh et al. 2002; Katoh and Standley 2013). After trimming sequences to identify the region of similarity, the alignment was visually analyzed using a phylogenetic tree constructed in Geneious Prime using FastTree v.2.1.11 (Price et al. 2009), with the outgroup polyclad worm, *Pseudobiceros bedfordi* (NCBI GenBank: KY421515).

## 3 Results

We designed a species‐specific assay to detect the target *Prosthiostomum acroporae*, which amplified a 192 bp fragment of the mtCOI gene region (Table 2). Primer‐BLAST analysis of the primer pair returned predicted amplicons of a few non‐target taxa including freshwater caddisfly ( *Agapetus fuscipes* ), Antarctic bony fish ( *Nansenia antarctica* ), plant species (*Petasites albus*), and another polyclad flatworm, *Prosthiostomum siphunculus* . All non‐target matches have mismatches greater than four, except for *Agapetus fuscipes* , which had three mismatches but does not overlap with *P. acroporae* range or habitat. Further investigation of the non‐target flatworm match *P. siphunculus* revealed one mismatch at the 3′ ends of primers, reducing the likelihood of efficient amplification under assay conditions (Simsek and Adnan 2000), and BLASTn of the probe sequence showed no high identity matches in this non‐target species. As all non‐target matches displayed either at least four mismatches, had low percent identity to the probe sequence, or did not occur within target species range, the assay was considered specific to *P. acroporae*.

**TABLE 2:** Primers and probes designed from the detection of *Prosthiostomum acroporae*.

Name	Sequence (5′‐3′)	Length (bp)
AEFW‐315F	CTTTGTWAGAAAAGGAGTAGGAGG	24
AEFW‐506R	CGRTATCATGCCATTCCCCT	20
AEFW‐385P	GGAAGWAGTGTTGATTTAGCAATATTT	27

ddPCR optimisation resulted in a 25 μL reaction volume containing the following: 12.5 μL Bio‐Rad ddPCR Supermix for Probes (no dUTP), 900 nM each of forward and reverse primers, 100 nM of a hydrolysis TaqMan probe, 5 μL DNA template, and PCR‐grade pure water (Thermo Fisher Scientific) to volume (Appendix S2). Annealing temperature optimization resulted in thermal cycling consisting of 95°C for 10 min; 40 cycles of 94°C for 30 s and 58°C (Appendix S3) for 1 min, followed by an enzyme deactivation step of 98°C for 10 min, and held at 4°C until signal reading.

### 3.1 Assay Sensitivity and Specificity

The dilution series showed an exponential decrease in DNA concentrations and exhibited high linearity (*R* ^2^ = 0.975, *p* < 0.0001) (Appendix S4). Amplification of *P. acroporae* DNA was successful in all replicate tests down to 0.076 pg DNA per ddPCR reaction, or 2.3 (±1.1 SD) copies mtCOI gene per ddPCR reaction (Table 3, Appendix S4). Thus, the limit of detection (LOD) for this *P. acroporae* assay was defined as 0.076 pg DNA or 2.3 copies per ddPCR reaction. The ddPCR assay sensitivity reduced to 50% at 0.038 pg per ddPCR and to 0% at further dilutions (Table 3). Based on the 35% CV threshold, the LOQ was determined as 0.76 pg. DNA per ddPCR reaction, or 47.13 (±11.41 SD) copies per ddPCR reaction.

**TABLE 3:** Results from a dilution series of *P. acroporae* gDNA using digital droplet PCR (ddPCR).

*P. acroporae* concentration (pg/mL^−1^)^a^	Total DNA (pg) per ddPCR	ddPCR mean copy per reaction	SV	%CV	% positive replicates^b^
3.80	19	1828.46	48.71	2.66%	100%
0.76	3.8	242.08	13.70	5.67%	100%
0.152	0.76	47.13	11.41	24.27%	100%
0.0304	0.152	6.67	3.12	47.32%	100%
0.0152	0.076	2.26	1.1	52.31%	100%
0.0076	0.038	1.12	1.46	131.20%	50%
0.0038	0.019	0.00	0	N/A	0%
0.0019	0.0095	0.00	0	N/A	0%
NTC	NTC	0	0	N/A	0%

Abbreviations: CV%, coefficient of variation; NTC, no‐template control; SV, standard variation.

^a^ DNA concentration of the 5‐fold dilution series was calculated based on measured values of a concentrated gDNA extract.

^b^ The data presented are from 8 replicates per dilution concentration.

**FIGURE 2:**
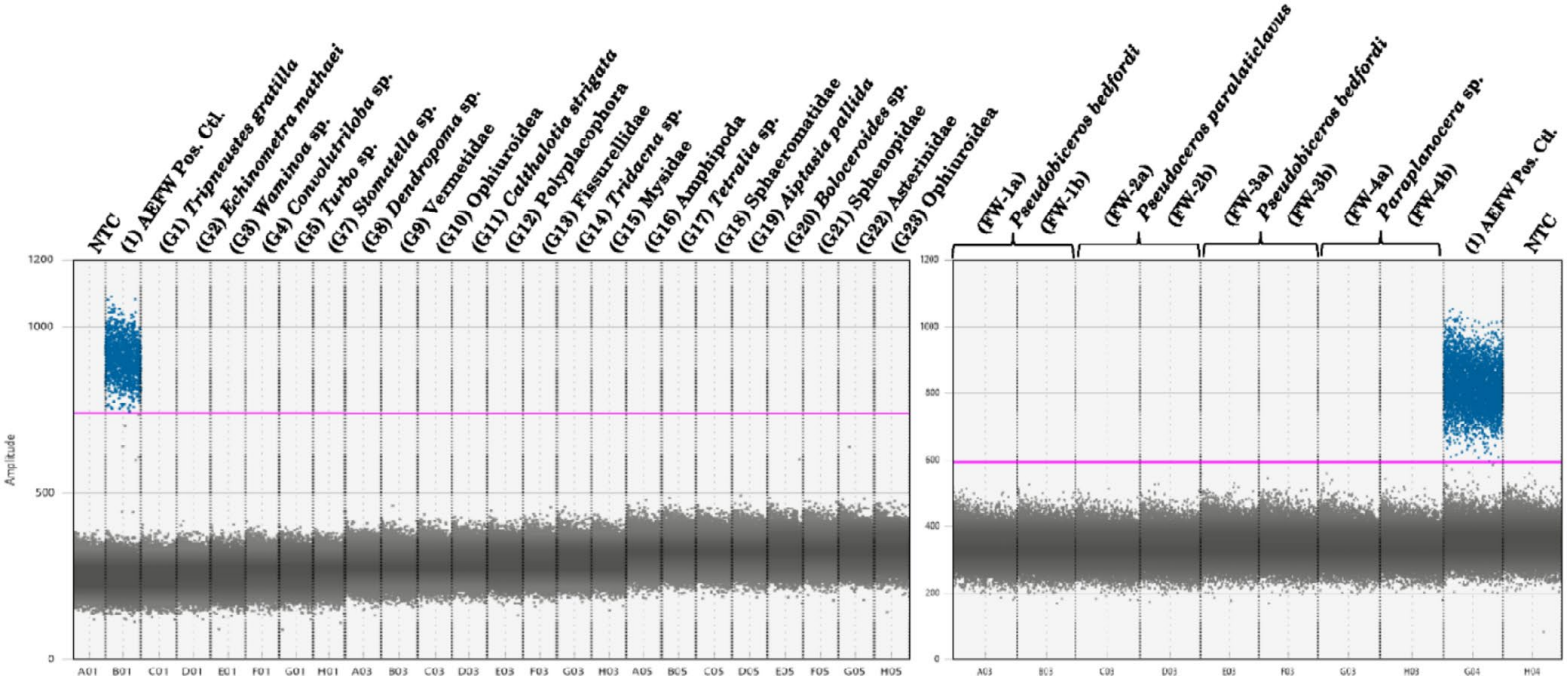
Specificity against non‐target species gDNA via droplet digital PCR assay. Left panels contain species of common occurring aquarium invertebrates, labeled at lowest possible taxonomic resolution. Right panel shows closely related polyclad flatworm species. Specimen information for non‐target species in both panels is listed in Appendix S1. The number in bracket refers to specimen ID (Table 1, Appendix S1). The pink line indicates the threshold for positive droplets set based on the positive droplet clustering of the positive control *P. acroporae* (“AEFW”, specimen ID 1, Table 1). All samples were tested in ddPCR technical replicates. NTC, no template control.

Assay in vitro specificity testing resulted in no amplification of any non‐target species (Appendix S1) above the positive control threshold (Figure 2), indicating that the assay is specific for the detection of *P. acroporae* DNA. All commonly occurring aquarium species and closely related species did not cross‐amplify. The no template negative controls (NTC) included with the ddPCR assay sensitivity and specificity testing showed no positive amplification.

### 3.2 eDNA Application to Coral Aquaculture

*P. acroporae* eDNA was detected on 7th November in the *Acropora millepora* tank at a concentration of 1.5 ± 0.6 (mean ± SD) copies per ddPCR reaction. Although this value was below the assay's LOD (2.3 ± 1.2 copies per ddPCR reaction), the difference from the LOD was not statistically significant (*p* = 0.276), and therefore the detection was interpreted as positive, indicative of early presence (Table 4). At the sequential sampling point 5 days later (12th November), the mean concentration was 0.5 ± 0.5 copies per ddPCR reaction, well below the LOD (*p* < 0.05), and thus considered negative. eDNA samples collected from *A. millepora* tank on 5 and 14 December 2023 both showed positive detection of *P. acroporae* eDNA above the LOD (Table 4), however there was no visual confirmation for the presence of *P. acroporae*. In contrast, eDNA samples from the adjacent *A. spathulata* tank, tested negative for *P. acroporae* eDNA at all sampling points despite the shared sump with *A. millepora* , and there was no visual observation of *P. acroporae* on the coral colonies.

**TABLE 4:** Results from *P. acroporae* (AEFW) eDNA testing in broodstock tanks.

Coral broodstock species	Date	Mean copies per ddPCR	SD	AEFW detection^a^	*p* ^b^
*A. millepora*	07‐Nov‐23	1.5	0.6	Yes	0.287*
	12‐Nov‐23	0.5	0.5	No	0.02
	5‐Dec‐23	5.1	5.2	Yes	0.198*
	14‐Dec‐23	3.0	0.6	Yes	0.274*
*A. spathulata*	5‐Dec‐23	0.7	0.7	No	0.039
	14‐Dec‐23	0	0	No	N/A
*A. loripes*	8‐Dec‐23	0	0	No	N/A
	14‐Dec‐23	3.2	4.9	Yes	0.634*

*Note:* Mean copies of AEFW DNA per ddPCR reaction presented along with standard deviation (SD). A simplified detection metric (yes/no) based on the limit of detection is also provided.

^a^ Positive AEFW detection was defined as the mean at or above the limit of detection (LOD).

^b^ *T*‐test was conducted to compare the mean sample concentration with the LOD concentration (significance level 0.05).

* Mean ddPCR copy numbers are not statistically different from the limit of detection and suggest the presence of AEFW (LOD).

*P. acroporae* eDNA was detected in the *Acropora loripes* broodstock tank on the 14th of December. No detection was observed in this tank 6 days earlier, suggesting an increase in *P. acroporae* biomass over that period. This detection was corroborated by aquaculture technicians, who reported visually observing AEFW egg masses in the *A. loripes* holding tank on the same day as eDNA sampling (Table 4), although these observations were not quantitatively recorded. All negative controls, including inlet filtered seawater, DNA extraction controls, and no‐template controls (NTC) returned with negative ddPCR results for *P. acroporae* eDNA.

Agarose gel electrophoresis of eDNA amplicons from ddPCR‐positive *A. loripes* and *A. millepora* tanks showed the same molecular weight as the *P. acroporae* gDNA (~192 bp; Appendix S6) albeit smaller amplicons visible across coral‐tank samples (< 100 bp), indicating that the eDNA assay could detect *P. acroporae* eDNA in coral aquaculture tanks. Sanger sequencing of these ddPCR‐positive eDNA samples (*n* = 12, Table 4) retrieved six high quality DNA sequences, with at least one replicate per positive sampling timepoint having high quality DNA for analysis (1/4 replicates of *A. millepora* tank from December 5th 2023, 1/4 replicates of *A. millepora* tank from December 14th 2023, and 4/4 replicates of *A. loripes* tank from December 14th 2023). The MAFFT alignment of these sequences revealed that positive eDNA samples from the *A. millepora* and *A. loripes* host tanks fell within separate phylogenetic clades. All eDNA sample sequences from both broodstock tanks showed 76%~99% sequence similarity to the *P. acroporae* gDNA sequences from specimens 3, 4, 5, and 6, while they shared 51%~58% identity to the *P. acroporae* gDNA specimens 1 and 2 collected from an *Acropora millepora* colony sourced from Martin's Reef (Appendix S7, Table 1). This revealed that the *P. acroporae* gDNA specimens 1 and 2, forming a clade with the *Prosthiostomum hibana* and other *Prosthiostomum* species, were potentially genetically distinct from other *P. acroporae* in this study (Appendix S7).

## 4 Discussion

The developed ddPCR primer pair set, containing forward primer AEFW‐315F and the reverse primer AEFW‐506R, with the addition of the hydrolysis probe AEFW‐385P, successfully amplified a 192 bp fragment of the *P. acroporae* mtCOI gene through in silico, in vitro, and in situ testing. Through implantation in the aquaculture setting, the ddPCR assay proved its effectiveness to detect *P. acroporae* eDNA associated with infestations within adult *Acropora* sp. coral holding tanks. The final primer and probe concentrations of the optimized ddPCR assay were 900 and 100 nm respectively, with an annealing/extension temperature optimum for the 2‐step ddPCR of 58°C. The LOD of the ddPCR assay was found to be 0.076 pg. DNA (2.3 ± 1.1 copies) per ddPCR reaction, and the assay's specificity to *P. acroporae* was demonstrated when evaluated against non‐target flatworm species and other marine invertebrates occurring in aquaculture facilities through in silico and in vitro testing. Through implantation in the aquaculture setting, the ddPCR assay proved its effectiveness to detect *P. acroporae* eDNA associated with infestations within adult *Acropora* sp. coral holding tanks. Previously, Thalinger et al. (2021) developed a standardized eDNA assay validation scale ranging from Level 1 (Incomplete) to Level 5 (Operational), on which scoring scale our assay achieved a Level 4 (Substantial) as it meets its three minimum criteria—determination of the LOD, advanced in vitro testing, and multiple field samples (Thalinger et al. 2021). To reach Level 5 classification, our assay must undergo additional validation, including statistical modeling of detection probabilities and testing of environmental factors affecting eDNA detection.

Testing for *P. acroporae* eDNA in tanks holding broodstock corals demonstrated the assay capability to detect the presence or absence of *P. acroporae* eDNA in trace amounts, depending on timepoints and sampled sites. For example, in December 2023, eDNA sampled from the *A. millepora* tanks indicated positive presence of *P. acroporae* at two sampling timepoints, while the previous two tests in November 2023 respectively indicated an inconclusive detection (i.e., statistically significant but below LOD) and a negative detection. Notably, *A. millepora* colonies were relocated to a different tank within the same aquaculture system between November and December, suggesting that the results reflect temporal and/or spatial patterns of the *P. acroporae* eDNA presence. On December 5th, 2023, *Acropora spathulata* was introduced into the tank previously occupied by *A. millepora* in November, which is connected to the new tank housing *A. millepora* colonies with a shared sump for water recycling. Despite the partial recirculation between tanks and tank switch, eDNA samples from the *A. spathulata* tank were all below the assay LOD, while samples from the *A. millepora* tank consistently exceeded the assay LOD. It is important to note that samples were taken from water surrounding coral colonies from the aquaria component holding the two species separately. This spatial variation in detection suggests that our assay could reveal the localized presence of *P. acroporae* eDNA, likely reflecting the presence of *P. acroporae* in the *A. millepora* tank but not throughout the semi‐recirculated system. As predation of *P. acroporae* on *A. spathulata* has been previously documented (Barton et al. 2019), the flatworm's host specificity against *A. spathulata* is unlikely to explain this result, although further studies on host preference for *A. millepora* are needed for confirmation.

Similarly, no *P. acroporae* eDNA was detected in the *Acropora loripes* broodstock tank on 8 December 2023, but positive detection occurred 6 days later, on 14 December, indicative of an increase of the target eDNA over time. Notably, this tank was maintained in a propagation system with 100% flow‐through conditions (200 L/h), suggesting the presence of a higher *P. acroporae* biomass than in the recirculating systems housing *A. millepora* and *A. spathulata* as the eDNA retention is expected to be shorter in flow‐through tanks due to dilution and increased physical removal from continuous water exchange (Shogren et al. 2016).

Sequence analysis on positive eDNA samples confirmed homology and phylogenetic clustering with the gDNA derived from *P. acroporae* tissue samples used to develop the ddPCR assay, verifying the *P. acroporae* detection in the coral tanks (Appendix S7). This sequence analysis also suggested that two flatworm tissue specimens originating from *A. millepora* corals collected at Martin's Reef (Specimen ID 1–2, Table 1) were separate species from *P. acroporae* specimens collected from Falcon Reef, while one of the Martin Reef specimens (Specimen ID 3, Table 1) formed a clade with those from Falcon Reefs (Specimen ID 4–6, Table 1). Within the latter clade, two monophyletic clades were formed between the eDNA derived sequences from tanks holding *A. millepora* and *A. loripes* . Host–parasite specificity is not uncommon for corallivores and their hosts. For example, parasitic nudibranchs in the genus *Phestilla* (Fionidae: Trinchesiidae) shows high host specificity between coral species, with previous reports highlighting that host coral is an important driver in speciation within this genus (Faucci et al. 2007; Mehrotra et al. 2024; Ritson‐Williams et al. 2003). While there is less literature on prey specificity of polyclad flatworms, one example such as, *Maritigrella crozieri* has been observed to feed exclusively on the ascidian, *Ecteinascidia turbinate*, despite access to closely related ascidians (Le Newman et al. 2000). These examples, alongside our phylogenetic results, raise the possibility that *P. acroporae* may also have evolved a degree of host specialization, potentially at the *Acropora* species level. However, given the use of mitochondrial COI as the target region for this assay development and the small number of individuals used to develop the primers, it is prudent to not draw specific conclusions about whether the “*P. acroporae*” clade reflects a population genetic structure or interspecific divergence. These phylogenetic results were intended to give clarity to the placement of short eDNA sequences relative to gDNA sequences, and higher phylogenetic resolution is needed to investigate specific host‐flatworm associations. The literature to date is sparse on *Prosthiostomum* taxonomy particular from the Great Barrier Reef Australia (Rawlinson et al. 2011), and thus further studies are needed to characterize molecular systematics around *P. acroporae*. Ultimately, the intended advantage for development of this eDNA assay is to capture the phylogenetic diversity of *P. acroporae* and closely related lineages relevant to flatworm infestations in *Acropora* cultures. As additional genetic diversity within and around *P. acroporae* is uncovered, primer design may require refinement to minimize the risk of false negatives arising from sequence mismatches. Broadening primer coverage to target a wider phylogenetic range could improve inclusivity but may reduce assay specificity and increase the risk of off‐target amplification. Alternatively, the development of lineage‐specific assays may provide a practical approach to balancing target diversity with high analytical specificity.

One key advantage of the eDNA method is that aquarium sampling is fast and relatively easy, thus aquarists with little scientific background can be trained to collect samples. In our assay workflow, the subsequent laboratory procedures including DNA extraction and ddPCR can be labor intensive for numerous samples. Coral aquaculture operations in more realistic settings will require speed and scalability to keep pace with timely detection and potential mitigation of pest infestation. Improving workflows that seamlessly connect sampling to analysis is therefore key to effective management of these systems. For example, deploying automated eDNA sampling devices in coral broodstock tanks (Formel et al. 2021), streamlining or eliminating DNA extractions (Scriver et al. 2024), followed by the development of non‐PCR amplification assays, could enable on‐site detection of pests without the need for molecular approaches to be employed (Rieder et al. 2025; Yugovich et al. 2025). Rapid, point‐of‐care assay options include nucleic acid lateral flow assays (LFA), similar to COVID‐19 tests (Li et al. 2019; Thongkao et al. 2015; Ying et al. 2021), which use simplified DNA extraction methods and visualize products of isothermal amplification techniques such as loop‐mediated isothermal amplification (LAMP) (Aonuma et al. 2010) or recombinase polymerase amplification (RPA) (Jaroenram and Owens 2014). These approaches can also be combined with CRISPR‐Cas‐based detection platforms, such as RPA‐CRISPR‐Cas assays (Junior et al. 2025; Rieder et al. 2025; Williams et al. 2023, 2019), further enhancing sensitivity and general applicability.

This study provides a foundation for the application of ddPCR‐based eDNA detection of *P. acroporae* in coral aquaculture systems and identifies key opportunities for refinement. The reported limit of detection (LOD), established using purified genomic DNA under controlled conditions, offers a sensitivity benchmark. However, validation in operational tank water will further clarify assay performance in the presence of potential inhibitors such as coral mucus, dissolved organics, feed residues, biofilms, and high microbial loads. Additional controlled experiments linking known *P. acroporae* egg and adult abundance to eDNA concentrations in operational tank water will give greater insight to eDNA shedding dynamics while accounting for tank‐specific environmental factors including water turnover rates, flow dynamics, and UV exposure. While our sampling approach demonstrated initial assay validation, future studies should also optimize sampling parameters including location relative to host colonies and flow, water volume, and frequency, to maximize reliability across both flow‐through and recirculating systems. These next steps will enhance assay robustness for use in coral aquaculture operations and support its development toward a practical early‐warning tool.

When captive corals are to be deployed to wild reefs for restoration purposes or relocated through the marine ornamental trade, effective coral biosecurity is needed through the application of robust, cost‐effective diagnostic tools that can sensitively detect and prevent the spread of pests (Khodzori et al. 2024; Sheridan et al. 2013). As coral aquaculture grows to accommodate these sectors, it is critical that this growing industry adapts modern biosecurity diagnostic technologies such as eDNA. Our study demonstrates that a sensitive eDNA–ddPCR assay can be an effective tool for early detection of *P. acroporae* in coral aquaculture systems. Routine implementation of this approach could improve understanding of parasite population dynamics within aquaculture facilities, enabling proactive monitoring, tracking, and timely interventions against the pest. As we demonstrate in this study, the use of eDNA detection is a step forward in the development of a practical biosecurity toolbox for the industry.

## Author Contributions

**Clare M. Grimm:** data curation (equal), investigation (lead), methodology (equal), project administration (equal), validation (lead), writing – original draft (lead). **Jonathan Barton:** conceptualization (equal), methodology (equal), writing – review and editing (supporting). **David G. Bourne:** conceptualization (equal), funding acquisition (supporting), supervision (equal), writing – review and editing (lead). **Yui Sato:** conceptualization (equal), supervision (equal), writing – original draft (equal), writing – review and editing (equal). **Jason Doyle:** conceptualization (equal), formal analysis (equal), funding acquisition (equal), supervision (lead), validation (equal), writing – review and editing (lead).

## Funding

This work was supported by the Reef Restoration and Adaptation Program.

## Conflicts of Interest

The authors declare no conflicts of interest.

## Supporting information


**Appendix S1:** Non‐target organisms tested for specificity of the AEFW eDNA assay. Specimens FW 1–4 are non‐target platyhelminth flatworms collected from Heron Island in April 2023 (Desbiens et al. 2023). All other specimens collected from coral tanks the National Sea Simulator at the Australian Institute of Marie Science in August 2022. Specimens G1.2–G24 were collected opportunistically in coral tanks and identified to the lowest taxonomic resolution.
**Appendix S2:** For all annealing/extension temperature settings, the greatest separation of positive and negative ddPCR droplets, and therefore the highest signal‐to‐noise ratio was found using 900 nM primers and 100 nM probe (Appendix S2 Table, Appendix S2 Figure). At these primer/probe concentrations, the signal‐to‐noise ratio was slightly larger above annealing temperature of 60°C and below annealing temperature of 58°C, with an obvious reduction in “rain” (i.e., non‐specific ddPCR amplification signals below the amplitude of the positive threshold) between 58°C and 60°C (Appendix S3). At 58°C, the signal‐to‐noise ratio was 3.04 while 2.90 at 59°C. When we examined the coefficient of variation (%CV) of the positive droplet fluorescence of this primer probe combination, we found the lowest %CV in annealing/extension temperatures of 58°C and 59°C, indicating that these temperatures provide the smallest variation in positive amplification fluorescence intensity.
**Appendix S3:** AEFW ddPCR assay optimization. The combined annealing/extension temperature cycle for each primer/probe combination (e.g., 900/100 nM) was tested in 1°C increments from 57°C to 62°C (left to right). The image below shows an example from duplicate reactions.
**Appendix S4:** The standard curve of the dilution series (top) was fitted using an exponential model with a concentration of *P. acroporae* COI (copies/μL) measured by ddPCR on the *y*‐axis against the log‐transformed value of the loaded DNA (ng) of the dilution concentrations on the *x*‐axis. The quantitative linearity of the dilution series (bottom) was assessed by plotting the log_10_‐transformed *P. acroporae* COI copy concentration measured by ddPCR plotted against the corresponding log_10_‐transformed inputted ng of DNA and fitted with linear regression. The equation of the linear, the goodness of fit (*R* ^2^) and the associated *p*‐value are included in the plots. Note different *y*‐axis scales of the two plots.
**Appendix S5:** Testing to determine the limit of detection (LOD) of the digital droplet PCR assay for AEFW identified by DNA dilution series. The panels across the *x*‐axis are individual PCR reactions containing the dilution series of AEFW DNA per (pg/mL^−1^; see Table 4). *Y*‐axis indicates fluorescence intensity of each nanodroplet within the PCR reaction (amplitude). The positive threshold (pink line) was set based on the separation of the positive droplet clustering (top blue dots) and negative droplets (bottom gray dots). Figure is a typical example out of eight replicates.
**Appendix S6:** 2% Agarose gel electrophoresis of eDNA samples from broodstock sampling on 14th December 2023 (Table 4). Lanes contain eDNA amplification, separated based on the amplicon size, with a molecular weight ladder for reference on both sides. Numbers are sample IDs associated with an internal sample labeling scheme. AEFW+ = “Acropora‐eating flatworm positive control” NTC = “no template control”.
**Appendix S7:** Phylogenetic tree of eDNA sequences (blue), AEFW gDNA sequences (green; Table 1), and GenBank sequences of *Prosthiostomum* sp. (orange) and outgroup (black). GenBank sequences accessed on January 16th, 2025. Phylogeny constructed in Geneious Prime using FastTree v.2.1.1 based on a MAFFT alignment.

## Data Availability

All raw data associated with this study can be found either in the main text or Supporting Information. All genomic sequences used in this research were uploaded to NCBI GenBank and accession numbers are referenced in the main text.
