# Supplementary material for: Addressing a Flat‐Out Problem: Environmental DNA (eDNA) Exposes Silent Infestations of Acropora ‐Eating Flatworms ( Prosthiostomum acroporae ) in Coral Aquaculture
Source: Ecol Evol. 2026 Apr 17;16(4):e73386. doi: 10.1002/ece3.73386 (PMC13090110; doi:10.1002/ece3.73386)
Supplement: Supplementary file 1 — Appendix S1: Non‐target organisms tested for specificity of the AEFW eDNA assay. Specimens FW 1–4 are non‐target platyhelminth flatworms collected from Heron Island in April 2023 (Desbiens et al. 2023). All other specimens collected from coral tanks the National Sea Simulator at the Australian Institute of Marie Science in August 2022. Specimens G1.2–G24 were collected opportunistically in coral tanks and identified to the lowest taxonomic resolution. Appendix S2: For all annealing/extension temperature settings, the greatest separation of positive and negative ddPCR droplets, and therefore the highest signal‐to‐noise ratio was found using 900 nM primers and 100 nM probe (Appendix S2 Table, Appendix S2 Figure). At these primer/probe concentrations, the signal‐to‐noise ratio was slightly larger above annealing temperature of 60°C and below annealing temperature of 58°C, with an obvious reduction in “rain” (i.e., non‐specific ddPCR amplification signals below the amplitude of the positive threshold) between 58°C and 60°C (Appendix S3). At 58°C, the signal‐to‐noise ratio was 3.04 while 2.90 at 59°C. When we examined the coefficient of variation (%CV) of the positive droplet fluorescence of this primer probe combination, we found the lowest %CV in annealing/extension temperatures of 58°C and 59°C, indicating that these temperatures provide the smallest variation in positive amplification fluorescence intensity. Appendix S3: AEFW ddPCR assay optimization. The combined annealing/extension temperature cycle for each primer/probe combination (e.g., 900/100 nM) was tested in 1°C increments from 57°C to 62°C (left to right). The image below shows an example from duplicate reactions. Appendix S4: The standard curve of the dilution series (top) was fitted using an exponential model with a concentration of P. acroporae COI (copies/μL) measured by ddPCR on the y‐axis against the log‐transformed value of the loaded DNA (ng) of the dilution concentrations on the x‐axis. The quantit [file ECE3-16-e73386-s001.docx]

**Appendices**

**Appendix S1.** Non-target organisms tested for specificity of the AEFW eDNA assay. Specimens FW 1 – 4 are non-target platyhelminth flatworms collected from Heron Island in April 2023 (Desbiens et al., 2023). All other specimens collected from coral tanks the National Sea Simulator at the Australian Institute of Marie Science in August 2022. Specimens G1.2-G24 were collected opportunistically in coral tanks and identified to the lowest taxonomic resolution.

| Specimen ID | Organism | Family | Phylum |  |
| --- | --- | --- | --- | --- |
| G1.2 | *Tripneustes gratilla* | Toxopneustidae | Echinodermata |  |
| G2.2 | *Echinometra mathaei* | Echinometridae | Echinodermata |  |
| G3 | *Waminoa* sp. | Convolutriloba | Xenacoelomorpha |  |
| G4 | *Convolutriloba* sp. | Convolutriloba | Xenacoelomorpha |  |
| G5 | *Turbo* sp. | Trochidae | Mollusca |  |
| G7 | *Stomatella* sp. | Trochidae | Mollusca |  |
| G8 | *Dendropoma* sp. | Vermetidae | Mollusca |  |
| G9 | Common Vermetid | Vermetidae | Mollusca |  |
| G10 | Common brittle star | Ophiuroidea | Echinodermata |  |
| G11 | *Calthalotia strigata* | Trochidae | Mollusca |  |
| G12 | Common Chitin | Polyplacophora | Mollusca |  |
| G13 | Keyhole limpet | Fissurellidae | Mollusca |  |
| G14 | *Tridacna* sp. | Cardiidae | Mollusca |  |
| G15 | Mysid Shrimp | Mysidae | Arthropoda |  |
| G16 | Amphipods | Amphipoda | Arthropoda |  |
| G17 | *Tetralia* sp. | Tetraliidae | Arthropoda |  |
| G18 | Small free-living isopod | Sphaeromatidae | Arthropoda |  |
| G19 | *Aiptasia pallida* | Aiptasiidae | Cnidaria |  |
| G20 | *Boloceroides* sp. | Boloceroididae | Cnidaria |  |
| G21 | Palythoa | Sphenopidae | Cnidaria |  |
| G22 | *Asterina* sp. | Asterinidae | Echinodermata |  |
| G23 | Brittle Star | Ophiuroidea | Echinodermata |  |
| G24 | *Acanthurus nigrofuscus* | Acanthuridae | Chordata |  |
| FW 1a/b | *Pseudobiceros bedfordi* | Pseudocerotidae | Platyhelminthes |  |
| FW 2a/b | *Pseudoceros paralaticlavus* | Pseudocerotidae | Platyhelminthes |  |
| FW 3a/b | *Pseudobiceros bedfordi* | Pseudocerotidae | Platyhelminthes |  |
| FW 4a/6 | *Paraplanocera* sp. | Planoceridae | Platyhelminthes |  |

**Appendix S2:** For all annealing/extension temperature settings, the greatest separation of positive and negative ddPCR droplets, and therefore the highest signal-to-noise ratio was found using 900nM primers and 100nM probe (Appendix S2 Table, Appendix S2 Figure). At these primer /probe concentrations, the signal-to-noise ratio was slightly larger above annealing temperature of 60°C and below annealing temperature of 58°C, with an obvious reduction in ‘rain’ (i.e. non-specific ddPCR amplification signals below the amplitude of the positive threshold) between 58°C and 60°C (Appendix S3). At 58°C, the signal-to-noise ratio was 3.04 while 2.90 at 59°C. When we examined the coefficient of variation (%CV) of the positive droplet fluorescence of this primer probe combination, we found the lowest %CV in annealing/extension temperatures of 58°C and 59°C, indicating that these temperatures provide the smallest variation in positive amplification fluorescence intensity.

**Appendix S2 Table:** Assay optimization using six primer/probe concentrations and temperature gradient. AEFW template gDNA containing approximately 15 pg/ml was used for assay optimization. Values reported is the signal to noise ratio which is the mean amplitude of positive droplets divided by the mean amplitude of negative droplets.

|  | **Primer/Probe conc (nM)** | | | | | |
| --- | --- | --- | --- | --- | --- | --- |
| **Temp** | *900/250* | *900/100* | *600/250* | *600/100* | *400/250* | *400/100* |
| **62** | 1.98 | 3.23 | 1.56 | 2.54 | 1.36 | 2.27 |
| **61** | 1.97 | 3.13 | 1.58 | 2.54 | 1.42 | 2.38 |
| **60** | 1.96 | 2.99 | 1.58 | 2.41 | 1.45 | 2.34 |
| **59** | 2.02 | 2.90 | 1.64 | 2.33 | 1.52 | 2.27 |
| **58** | 2.17 | 3.04 | 1.77 | 2.46 | 1.64 | 2.38 |
| **57** | 2.29 | 3.18 | 1.90 | 2.61 | 1.75 | 2.53 |

**Appendix S2 Figure.** Mean fluorescent amplitude of positive droplets, standard deviation, and coefficient of variation (%CV) for annealing/extension temperatures ranging from 57°C to 62°C.

| **Annealing/Extension Temperature (°C)** | **mean amplitude** | **SD** | **%CV** |
| --- | --- | --- | --- |
| **62** | 939.5 | 75.7 | 8.1 |
| **61** | 914.8 | 71.3 | 7.8 |
| **60** | 878.6 | 61.1 | 7.0 |
| **59** | 876.5 | 55.8 | 6.4 |
| **58** | 908.8 | 58.1 | 6.4 |
| **57** | 957.5 | 61.8 | 6.5 |


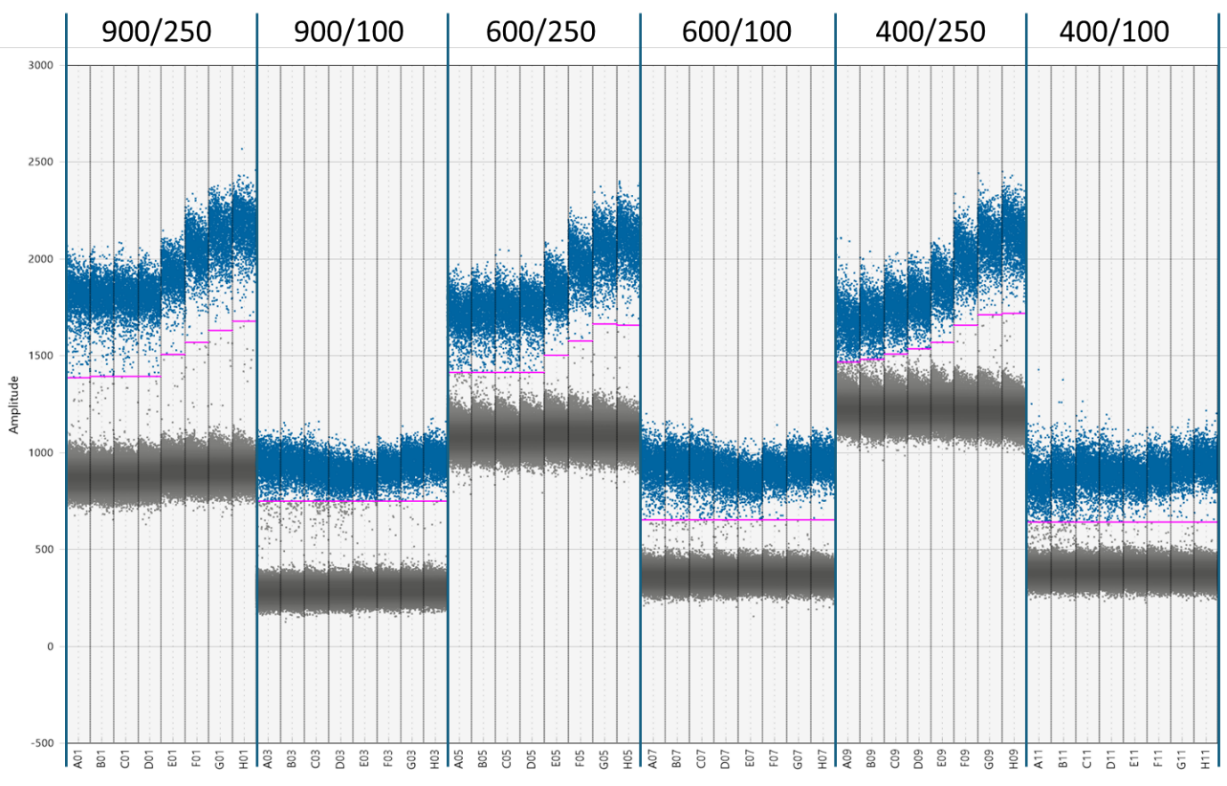


**Appendix S3.** AEFW ddPCR assay optimization. The combined annealing/extension temperature cycle for each primer/probe combination (e.g. 900/100 nM) was tested in 1°C increments from 57 – 62°C (left to right). The image below shows an example from duplicate reactions.


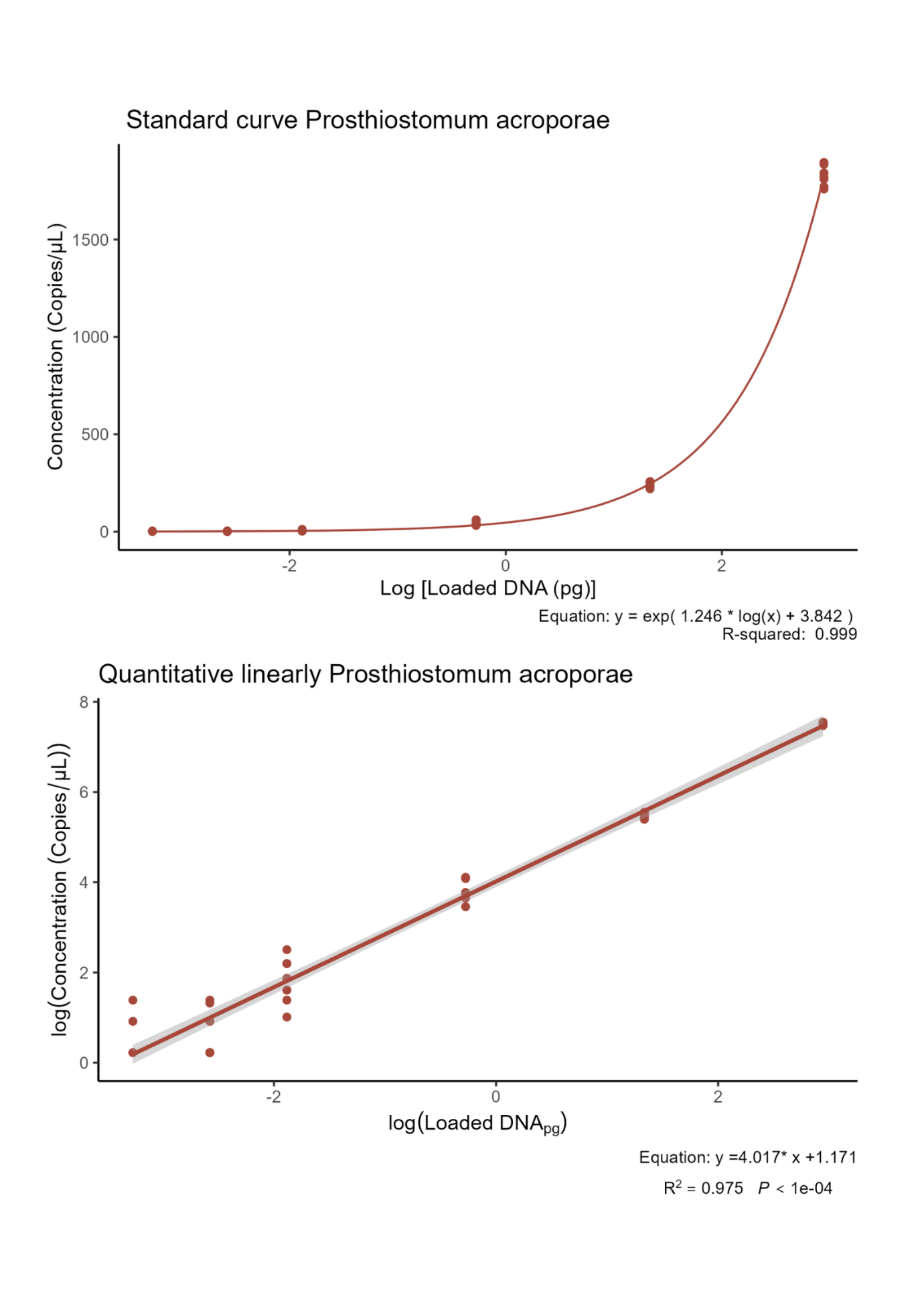


**Appendix S4:** The standard curve of the dilution series (top) was fitted using an exponential model with a concentration of *P. acroporae* COI (copies/µL) measured by ddPCR on the y-axis against the log-transformed value of the loaded DNA (ng) of the dilution concentrations on the x-axis. The quantitative linearity of the dilution series (bottom) was assessed by plotting the log10-transformed *P. acroporae* COI copy concentration measured by ddPCR plotted against the corresponding log10-transformed inputted ng of DNA and fitted with linear regression. The equation of the linear, the goodness of fit (R^2^) and the associated p-value are included in the plots. Note different y-axis scales of the two plots.


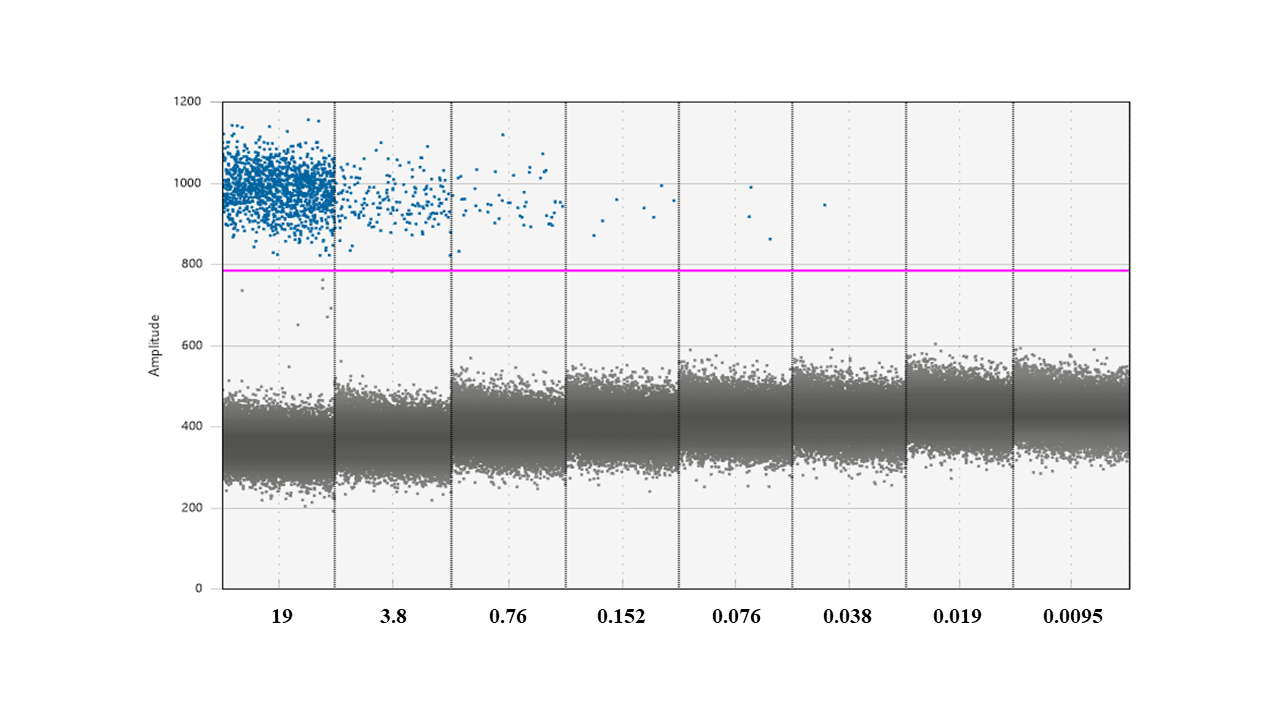


**Appendix S5:** Testing to determine the limit of detection (LOD) of the digital droplet PCR assay for AEFW identified by DNA dilution series. The panels across the x-axis are individual PCR reactions containing the dilution series of AEFW DNA per (pg/mL^-1^; see Table 4). Y-axis indicates fluorescence intensity of each nanodroplet within the PCR reaction (amplitude). The positive threshold (pink line) was set based on the separation of the positive droplet clustering (top blue dots) and negative droplets (bottom gray dots). Figure is a typical example out of eight replicates.


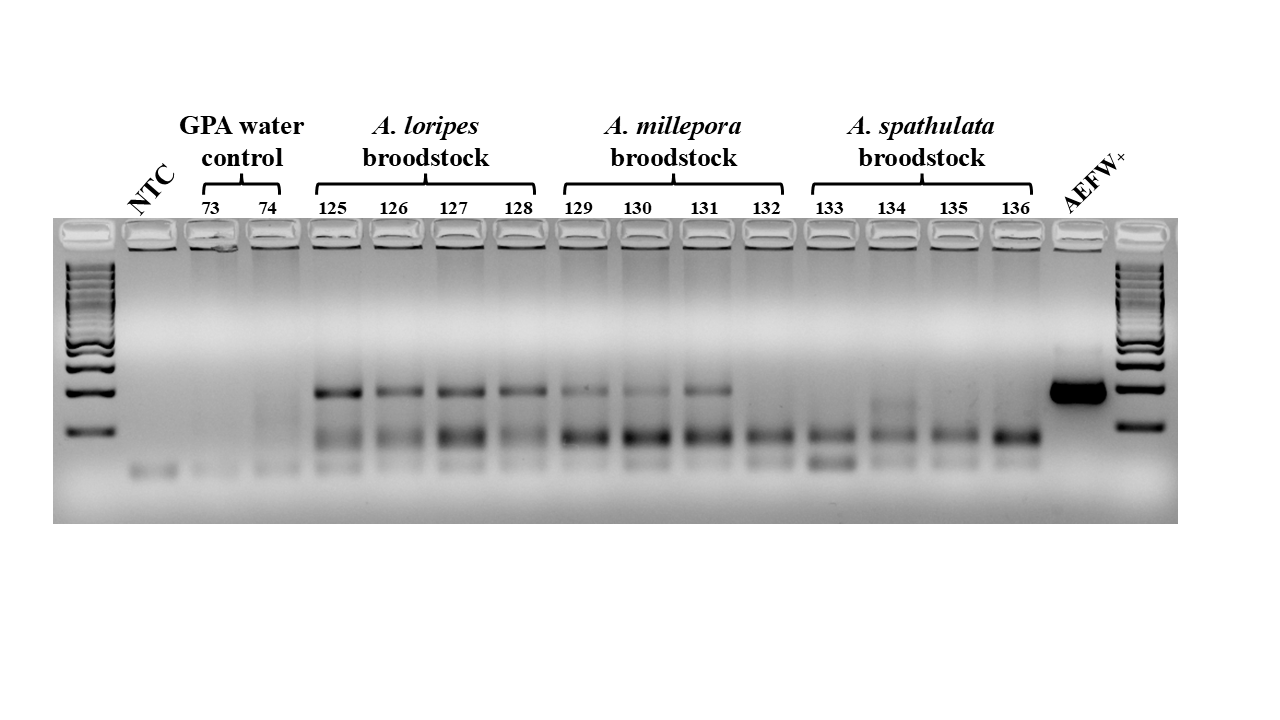


**Appendix S6:** 2% Agarose gel electrophoresis of eDNA samples from broodstock sampling on 14^th^ December 2023 (Table 4). Lanes contain eDNA amplification, separated based on the amplicon size, with a molecular weight ladder for reference on both sides. Numbers are sample IDs associated with an internal sample labeling scheme. AEFW+ = “Acropora-eating flatworm positive control” NTC = “no template control”


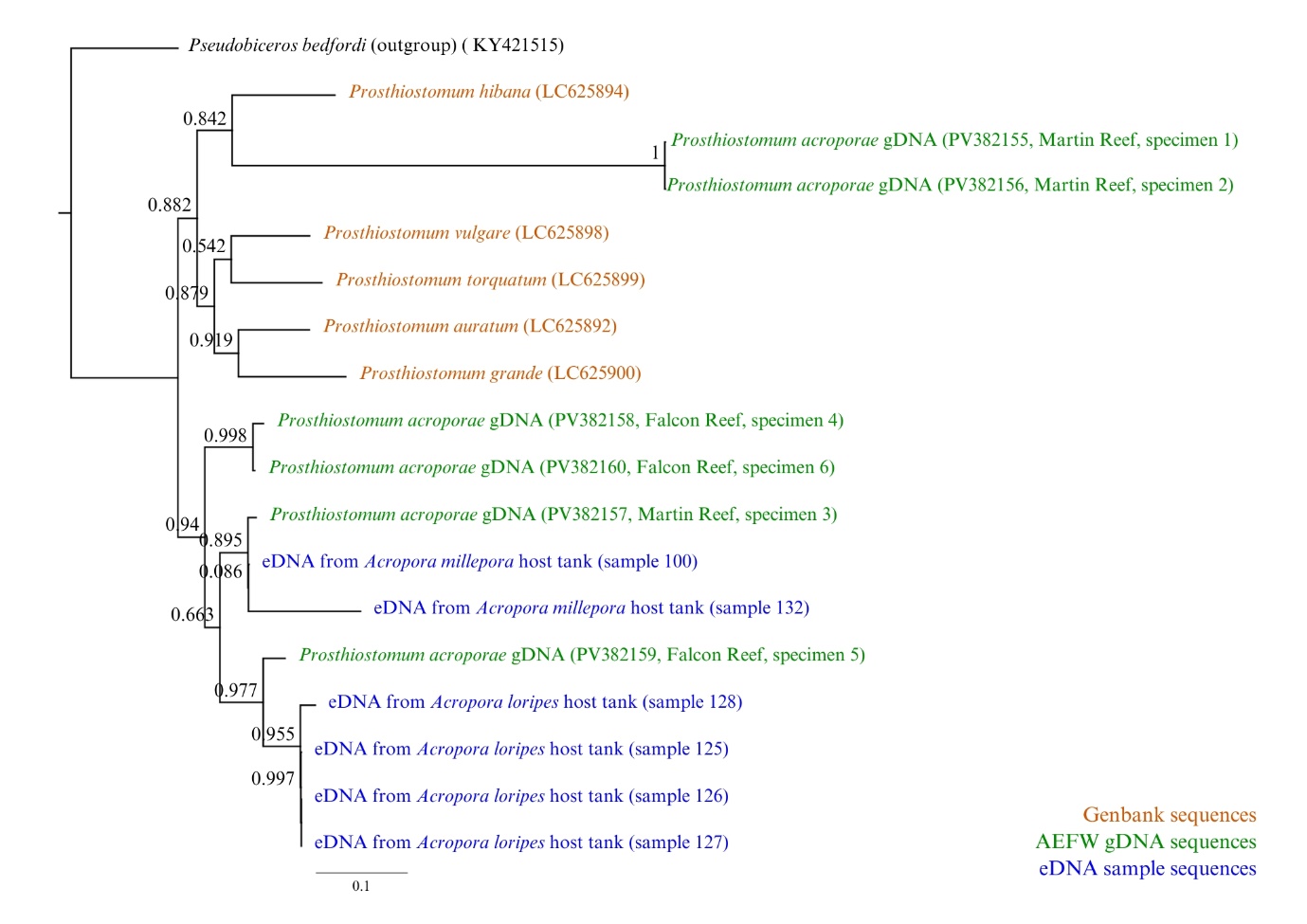


**Appendix S7**: Phylogenetic tree of eDNA sequences (blue), AEFW gDNA sequences (green; Table 1), and GenBank sequences of *Prosthiostomum* sp. (orange) and outgroup (black). GenBank sequences accessed on January 16^th^, 2025. Phylogeny constructed in Geneious Prime using FastTree v2.1.1 based on a MAFFT alignment.
